# Relationship between *rs6715345* Polymorphisms of *MIR-375* Gene and *rs4939827* of *SMAD-7* Gene in Women with Breast Cancer and Healthy Women: A Case-Control Study

**DOI:** 10.31557/APJCP.2020.21.8.2479

**Published:** 2020-08

**Authors:** Seyed-Mehdi Hashemi, Mohammad Hashemi, Gholamreza Bahari, Afsaneh Khaledi, Hoseinali Danesh, Abolghasem Allahyari

**Affiliations:** 1 *Clinical Immunology Research Center, Department Of Internal Medicine, Zahedan University of Medical Sciences, Zahedan, Iran. *; 2 *Hematology And Medical Oncology Ward, Ali-Ebne-Abitalelb Hospital, Zahedan University of Medical Science, Zahedan, Iran. *; 3 *Department of Clinical Biochemistry, School of Medicine, Zahedan University of Medical Sciences, Zahedan, Iran. *; 4 *GP, Zahedan University of Medical Sciences, Zahedan, Iran.*; 5 *Plastic, Reconstructive & Aesthetic Surgeon, Zahedan University of Medical Sciences, Zahedan, Iran. *; 6 *Department of Haematology and Medical Oncology, Mashhad University of Medical Sciences, Mashhad, Iran. *

**Keywords:** Genetic polymorphisms, SMAD7, MIR-375, breast neoplasm

## Abstract

**Background:**

Today, the role of microRNAs in the pathogenesis of breast cancer has been established. Genetic mutations play a significant role in determining the risk factors of cancer. The polymorphism of these two genes can alter their expression. This study has been performed to investigate the relationship between polymorphisms of *rs6715345* of *miR-375* gene and *rs4939827* of the *SMAD7* gene and development of breast cancer in a population in southeastern Iran.

**Methods:**

This case-control study was performed on the blood sample of 205 patients with breast cancer and 200 healthy individuals for investigating the *rs34917480* and* rs4939827* polymorphisms using the PCR-RFLP method. The data were analyzed by t-test, χ^2^, and logistic regression. The SPSS v18.0 used for data analysis.

**Results:**

The findings of this study indicated that the risk of developing breast cancer does not have a significant relationship with rs6715345 polymorphism of *miR-375* gene (p>0.05). However, the* rs4939827* polymorphism of the *SMAD7* gene was significantly linked to the risk of developing breast cancer in the southeastern population in Iran (p**<**0.05).

**Conclusion:**

The results suggest that the* rs4939827* polymorphism of the *SMAD7* gene can lead to an increased risk of incidence of breast cancer in the southeastern population in Iran.

## Introduction

Breast cancer is the most common cancer and the main cause of cancer-induced mortality among women worldwide whether in general or in less developed countries (DeSantis et al., 2019). According to the latest statistics of disease burden studies in 2019, it caused the death of more than 626,679 patients. It is also projected that by 2040, its prevalence would reach more than 46% (Pilevarzadeh et al., 2019). The risk of factors of breast cancer includes genetic, reproductive, and hormonal factors, the use of oral contraceptives, and having no children (Takalkar et al., 2014). Also, breast cancer during younger ages increases its chance of mortality (Zehentmayr et al., 2016). Clinically, breast cancer is considered a heterogeneous disease; although all of them have similar manifestations, they differ molecularly and in terms of expression of genes, response to conventional treatments, and prognosis. A conventional classification that has important clinical applications is based on the expression of *HER2 (ErbB-2)*, estrogen receptor (ER), and progesterone receptor (PR) (Perou et al., 2000). The overexpression of the *ErbB-2* gene, which encodes HER2 oncoprotein exists in 20-25% of breast cancers, which is associated with poor prognosis, the higher chance of metastasis, and relapse (Venturutti et al., 2016). Accordingly, the human anti-HER2 antibody, trastuzumab (Herceptin) has been used for treating HER2-positive cells (Slamon et al., 2001). Nevertheless, therapeutic response to monotherapy with trastuzumab in this type of breast cancer is less than 35%, and most patients with this type of treatment become resistant to this drug after initial response (Ye et al., 2014). Despite recent advances in adjuvant therapies such as hormone therapy, chemotherapy, and biological treatment, the prognosis of breast cancer is still poor (Pickle et al., 2007). For this reason, molecular properties and genetic susceptibility are very important factors that determine the prognosis of breast cancer, the extent of invasion and tumor relapse, as well as response to treatment (Lazaridis et al., 2014). For example, 70% of invasive cases of breast cancer show overexpression of ER- α receptor, which is known as an early process in tumorigenesis and progression of breast cancer (Simonini et al., 2010). Accordingly, in recent years, numerous studies have focused on identifying biomarkers for early diagnosis, prediction of response to treatment, and prognosis, where microRNAs have especially attracted attention (Nguyen-Dien et al., 2014). MicroRNAs (miRNAs) are a group of endogenous noncoding RNAs which are 18-23 nucleotides long and regulate the gene expression across the surface following transcription (Ambros, 2004). Thus, they can affect many biological processes and activities of cells including cell cycle, differentiation, growth, and metabolism (Kong et al., 2012). It has been found that miRNAs play a significant role in many human diseases especially cancers – whether as an oncogene or as a tumor inhibitor (Bartel, 2009). miRNAs control an extensive part of the human genome (Bartel, 2009), and it has been estimated that so far around 1,000 human miRNAs have been found(Friedman et al., 2009). Biogenesis of miRNAs begins from the nucleus and continues in the cytoplasm. Most miRNAs have been synthesized out of an initial transcript, including one head and one polyA tail transcribed by RNA polymerase II from the *miRNA* gene. The initial miRNA is broken by RNAse III Drosha–DGCR8 in the nucleus and Dicer in the cytoplasm to mature 22-nucleotide miRNAs (Babashah and Soleimani, 2011)(28). These single-stranded molecules regulate major biological processes across the surface following transcription through binding to the sequence of the target mRNA complement or inhibiting its translation (given the extent of mRNA and miRNA complement sequence) (Zhang et al., 2014).

Extensive evidence suggests that miRNAs are mal-expressed in a large number of human cancers (Farazi et al., 2013). Some of them act as an oncogene (oncomiR), while others as a tumor inhibitor, given which gene or pathway they affect (Zhang et al., 2014). Also, it has been found that miRNAs are also involved in metastasis (Edmonds et al., 2009). Recently, the relationship between miRNAs and hormonal receptors has attracted a great deal of attention (Di Leva et al., 2013). The Discovery of miRNAs has opened a novel way for diagnosis and treatment of cancer. miRNAs play a significant role in the regulation of tumor proliferation, metastasis, and invasion, and can be a potential target for cancer treatment and interventions (Lee and Dutta, 2009). In particular, they can be used as a non-invasive serum biomarker for the diagnosis of cancers (Chen et al., 2008). However, considering the population differences in the genome sequence and the presence of gene polymorphisms, further studies are required in this regard.

Single-nucleotide polymorphism (SNP) refers to single-nucleotide variations in the DNA sequence, causing the development of different genotypes. This can result in the development of population discrepancies with regards to the pathogenesis of diseases such as cancer with a special genotype (Wang et al., 1998). Thus, a better understanding of polymorphisms can support better treatment of breast cancer. This study was performed to investigate the relationship between *rs6715345* and *rs4939827* polymorphisms of *miR-375* and *SMAD-7 *genes respectively in women with breast cancer as well as in healthy women.

## Methods and Materials

*Study Design*

This case-control study was performed on women with breast cancer and on healthy women, who referred to an educational hospital affiliated with Zahedan University of Medical Sciences from 1 March 2019 to 30 August 2019. In this study, 205 samples belonging to breast cancer patients were investigated along with 200 samples of the control group who were healthy individuals. This disease of breast cancer patients was confirmed by pathology, while the healthy subjects had no history of the breast-associated disease. The samples were chosen thought a simple and available sampling method. Written and oral consent was taken from all participants. This study has been confirmed in the ethics committee of Zahedan University of Medical Sciences (IR.ZAUMS.REC.1396.325). STROBE checklist was used for reporting of the paper (Von Elm et al., 2008).

*Data collection*

To investigate the gene polymorphism, blood samples were taken from participants (5 ml) and collected in EDTA-containing tubes, and kept at -20ᵒC. The DNA was purified via standard salting out procedure (Hashemi et al., 2010). Specific primers for determining the gene polymorphism were designed via Restriction Fragment Length Polymorphism-PCR (RFLP-PCR) method for detecting single-nucleotide polymorphisms of *rs493982*7 and *rs6715345* belonging to SMAD-7 and miR-375 genes, respectively (Table 1).

Next, the genotyping of these two polymorphisms was done by PCR. Here, 0.20 of the reaction solution containing 1 mcL of DNA, 1 mcL of forwarding and reverse primers, and 10 mcL of Taq Premix, as well as 7 mcL of sterilized distilled water with no DNAse, was used. PCR conditions were optimized as follows: 1) preheating for 5 min at 95ᵒC for the primary denaturation step, 2) Thirty 30-sec cycles at 95ᵒC, 3) 59ᵒC (the selected annealing temperature) for 30 s and 72ᵒC for 30 s, and 4) the extension step for 5 min at 72ᵒC. In the next step, the PCR product was used via the limiting enzymes for each polymorphism (Table 1-3) so that the sequences of interest would be created (Burtis et al., 2012). The tubes were placed in Bain Marie at the activity temperature specified for every enzyme and within the duration predetermined in the protocol, to provide the conditions for the activity of enzymes. Eventually, the digestion product of DNA pieces was electrophoresed on agarose gel 2%. Finally, the alleles of the intended polymorphisms were observed through ethidium bromide staining.

*Data analysis*

For data description, descriptive tests (percentage, frequency, mean) were used. The disease characteristics were compared using χ2 and independent t-test. To determine the relationship between polymorphisms and breast cancer, logistic regression was used along with odds ratio (OR) calculation and confidence interval (CI) 95%. SPSS Version 18.0 for Windows (SPSS Inc., Chicago, IL, USA) was used to analyze the data. The confidence interval of 95% and a significance level of P-value less than 0.05 was considered significant.

## Results

**Figure 1:**
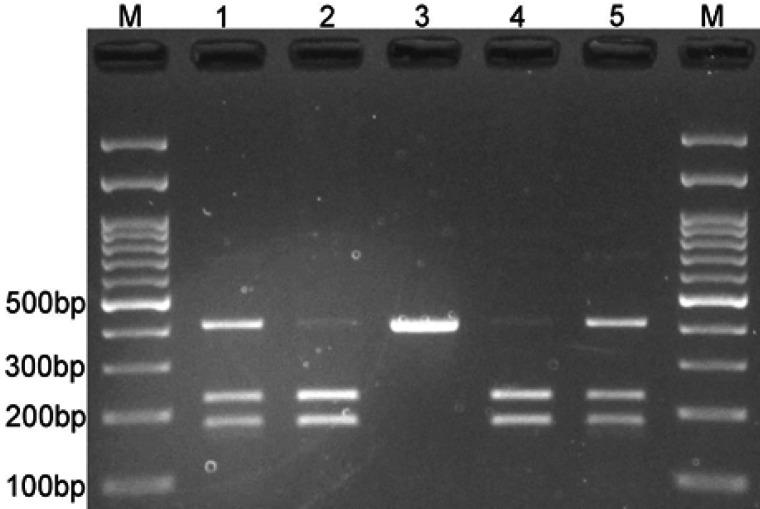
The Band of Genotypes CG (1), GG (2), and CC (3) in rs6715375 of *miR-375* Gene on Agarose Gel Following Electrophoresis

The mean age of women with breast cancer and healthy women was 48.56±10.67 and 48.41±11.29, respectively. The two groups did not differ significantly from each other statistically (p=0.894). The frequency of rs6715345 polymorphism of the *miR-375* gene was examined in 205 patients with breast cancer and 225 healthy women, and the genotypes were compared. The results indicated that there is no relationship between hereditary models as well as alleles of rs6715375 polymorphism and the risk of developing breast cancer (p>0.05) (Table 2). The pattern of electrophoresis of rs6715345 polymorphism on agarose gel with a band length of 226 and 179 base pairs for GG genotype and band length of 405 base pairs for CC genotypes have been shown in Figure 1.

**Figure 2:**
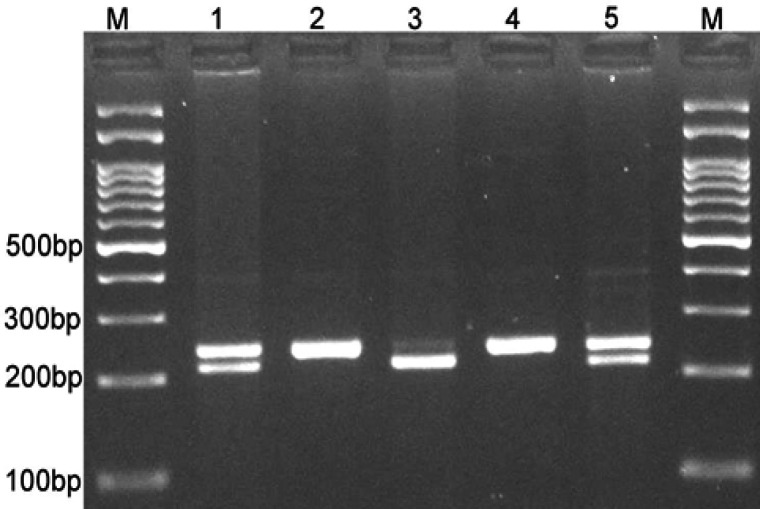
The Band of Genotypes of *TC (1), TT (2),* and *CC (3)* in *rs4939827* of *SMAD-7* Gene on Agarose Gel Following Electrophoresis

Also, regarding the frequency of rs4939827 polymorphism of *SMAD-7* gene and its genotypes in women patients with breast cancer and healthy women, it was found that the codominant hereditary models (OR=1.71, 95%CI=1.10-2.70, p=0.023) in comparison with TT (OR=2.22, 95%CI=1.20-4.26, p=0.017), CC in comparison with TT and dominant (OR=1.80, 95%CI=1.17-2.76, p=0.009, CT+CC in comparison with TT) and allele C (OR=1.44, 95%CI=1.08-1.89, *P*-value=0.013, allele C in comparison with allele T) were significantly related to the risk of developing breast cancer. The electrophoresis pattern of rs4939827 polymorphism on agarose gel with the band length of 242 base pairs for genotype TT and band length of 28 and 218 base pairs for genotype CC have been shown in Figure 2.

**Table 1:** Specific Primers for Determining Gene Polymorphism via RFLP-PCR Method for Detecting Single-Nucleotide Polymorphisms of *rs4939827* and *rs6715345* Belonging to SMAD-7 and *miR-375* Genes, Respectively

Gene polymorphism	Primer sequence (5`->3`) 4	Method	Restriction Enzyme	Fragment (bp)
*SMAD-7 rs4939827*	F: ACAAGCCTAAGATAAAAGGGGACT	PCR-RFLP	TaaI	T allele=242
	R: GAGTCTGAGGGAGCTCTGGGGTAC			C allele=218+24
*miR-375 rs6715345*	F: CCCGTATTACGACGCAGAA	PCR-RFLP	NaeI	C allele=405
	R: ACGTGTCAGCCGCAGAT			G allele=226+179

**Table 2:** The Frequency of Alleles and Genotypes of *rs6715375* Polymorphism of *miR-375* Gene and Their Comparison in the Case and Control Groups

*miR-375 rs6715345 G>C*	Breast cancer group N (%)	Healthy women N (%)	OR (95%CI)	*P*-value
Codominant				
GG	164 (80.0)	164 (82.0)	1	-
CG	29 (14.1)	32 (16.0)	0.91 (0.52-1.57)	0.781
CC	12 (5.9)	4 (2.0)	3.00 (0.95-8.63)	0.071
Dominant				
GG	164 (80.0)	164 (82.0)	1	-
CG+CC	41 (20.0)	36 (18.0)	1.14 (0.70-1.86)	0.615
Recessive				
CG+GG	193 (94.1)	196 (98.0)	1	-
CC	12 (5.9)	4 (2.0)	3.05 (0.97-8.75)	0.071
Allele				
G	357 (87.1)	360 (90.0)	1	-
C	53 (12.9)	40 (10.0)	1.34 (0.86-2.07)	0.225

**Table 3:** The Frequency of Allele and Genotypes of* rs4939827* Polymorphism of *SMAD-7 *Gene and Their Comparison in the Case and Control Groups

*SMAD-7 rs4939827 T>C*	Case (breast cancer) N (%)	Control (healthy women) N (%)	OR (95%CI)	*P*-value
Codominant				
TT	48 (23.4)	71 (35.5)	1	-
CT	121 (59.0)	105 (52.5)	1.71 (1.10-2.70)	0.023
CC	36 (17.6)	24 (12.0)	2.22 (1.20-4.26)	0.017
Dominant				
TT	48 (23.4)	71 (35.5)	1	-
CT+CC	157 (76.6)	129 (64.5)	1.80 (1.17-2.76)	0.009
Recessive				
CT+TT	169 (82.4	176 (88.0)	1	-
CC	36 (17.6)	24 (12.0)	1.56 (0.90-2.73)	0.125
Allele				
T	217 (52.9)	247 (62.7)	1	-
C	193 (47.1)	153 (38.3)	1.44 (1.08-1.89)	0.013

## Discussion

Today, considering the progressive prevalence of breast cancer despite the medical advances in the early treatment of breast cancer and increasing the survival of patients with this cancer, the causes of breast cancer should still be more profoundly understood as a complex issue (Shieh et al., 2020). Recognizing the risk factors of breast cancer in different dimensions can help us in better understanding the methods of prevention and early treatment of breast cancer (McPherson et al., 2000). In recent decades, molecular and cellular technological advances have provided a suitable opportunity to obtain a better understanding of the molecular and cellular aspects of breast cancer and the role of genetic factors in it (Network, 2012). On the other hand, further, understanding of the human genome and its regulating factors have resulted in the development of new areas for examining human diseases including cancer at the gene level and the factors affecting the expression or suppression of genes. Meanwhile, the role of microRNAs is of interest in the development of cancers (Hannafon et al., 2016). These noncoding RNAs and their polymorphisms have a significant impact on the regulation of gene expression in humans and can be used as biomarkers in the early diagnosis of human diseases (Bertoli et al., 2015). The results of the present study indicated that there is no significant relationship between *rs6715345* polymorphism of the *MIR375* gene and breast cancer in the studied population. To the best of the authors’ knowledge, no similar study has been found with this polymorphism. Unlike the present study, the study by Zhang et al., (2018), who had examined the variation of the *MIR275* gene in patients with breast cancer, showed that this gene is associated with diminished risk of breast cancer. This difference can be due to the difference in the type of gene polymorphism in the two studies. Also, another study on populations suffering esophageal squamous cell carcinoma in Kazakh patients (Zhu et al., 2015) indicated that the variation in *rs6715345* polymorphism of *MIR375 *gene leads to diminished risk of developing breast cancer. Based on our findings,* rs4939827* polymorphism of the *SMAD-7* gene is significantly linked to the risk of developing breast cancer. SMAD proteins are considered as the main mediators in TGF-β signaling pathway, playing a significant role in tumorigenesis-associated processes. Various studies have shown the role of *SMAD-7 *in tumorigenesis of different types of cancer (Greenwood and Bruna, 2019). Unlike the results of the present study, research on Caucasian English and Scottish populations suggest that there is no relationship between rs4939827 polymorphism of *SMAD-7 *gene and developing breast cancer (Gibson et al., 2009; Scollen et al., 2011). This can be due to the differences in the study populations as well as the type of polymorphisms examined in the two studies. Also, the study performed by Xin Lin testing the relationship between three polymorphisms (rs12953717, rs4939827, and rs4464148) showed that rs4939827 polymorphism was not associated with breast cancer, and the only rs1295371 was linked to this cancer (Li et al., 2011). However, concerning other cancers, in line with the present study, research on colorectal cancer in Caucasian and Asian populations (Zhang et al., 2018), stomach and lung cancers in a western population (Li et al., 2011) has also shown that *SMAD-7* gene is associated with developing cancer. The study by Kirac showed that *rs4939827* polymorphism of the *SMAD-7* gene is associated with developing colorectal cancer in a Croatian population (Kirac et al., 2013). Meta-analysis studies also show a relationship between *rs4939827 *polymorphism and the risk of developing different types of cancer including colorectal and esophageal cancers (Hong et al., 2016; Huang et al., 2016; Zhao et al., 2017). The most important limitations of the present study were: low sample size which may affect the study power regarding the relationship. Another limitation was the identical race of participants, all being Iranian and chosen from one single province in the east of the country.

In conclusion, this study indicated that single-nucleotide rs4939827 polymorphism of the *SMAD-7* gene is associated with developing breast cancer. However, single-nucleotide rs6715345 polymorphism of the *miR-375* gene was not significantly correlated with the risk of developing breast cancer. The results of the present study suggest attention to genetic dimensions of disease for the population suffering breast cancer in earlier diagnosis and better treatment of diseases. For better detection of the existing relationship, studies with a larger sample size are suggested. 
